# Robotic Surgery in Gynecology: An Updated Systematic Review

**DOI:** 10.1155/2011/852061

**Published:** 2011-11-28

**Authors:** Lori Weinberg, Sanjay Rao, Pedro F. Escobar

**Affiliations:** Department of OB/GYN and Women's Health Institute, Cleveland Clinic, Desk A-81, 9500 Euclid Avenue, Cleveland, OH 44195, USA

## Abstract

The introduction of da Vinci Robotic Surgery to the field of Gynecology has resulted in large changes in surgical management. The robotic platform allows less experienced laparoscopic surgeons to perform more complex procedures. In general gynecology and reproductive gynecology, the robot is being increasingly used for procedures such as hysterectomies, myomectomies, adnexal surgery, and tubal anastomosis. Among urogynecology the robot is being utilized for sacrocolopexies. In the field of gynecologic oncology, the robot is being increasingly used for hysterectomies and lymphadenectomies in oncologic diseases. Despite the rapid and widespread adoption of robotic surgery in gynecology, there are no randomized trials comparing its efficacy and safety to other traditional surgical approaches. Our aim is to update previously published reviews with a focus on only comparative observational studies. We determined that, with the right amount of training and skill, along with appropriate patient selection, robotic surgery can be highly advantageous. Patients will likely have less blood loss, less post-operative pain, faster recoveries, and fewer complications compared to open surgery and potentially even laparoscopy. However, until larger, well-designed observational studies or randomized control trials are completed which report long-term outcomes, we cannot definitively state the superiority of robotic surgery over other surgical methods.

## 1. Background

Minimally invasive surgery (MIS) has revolutionized the management of gynecologic disorders over the last 30 years. However, the most substantial improvements have come with the advent of robotic surgery. Initially, traditional laparoscopy afforded less invasive approaches to hysterectomies, tubal ligations, adnexal surgery, and even lymphadenectomies and radical hysterectomies. However, not all surgeons are comfortable with the laparoscopic approach due to its steep and extended learning curve, nor are all patients and procedures amenable to traditional laparoscopy. In fact, the majority of advanced gynecologic surgeries are still being performed through an abdominal incision. A recent study looking at the rates of open versus vaginal or laparoscopic hysterectomies from 2000 to 2005 at teaching and nonteaching hospitals in Illinois revealed that teaching hospitals were significantly less likely to perform abdominal hysterectomies (OR 0.69) after adjusting for confounding variables, but the overall rates of abdominal hysterectomies at teaching and nonteaching hospitals were still 82% and 77%, respectively [1]. Also, the rates of complications from laparoscopic hysterectomies were less than the rates from vaginal or abdominal hysterectomies. It has been well established that laparoscopic surgery has several advantages over abdominal surgery including shorter hospital stays, faster recoveries, less blood loss, better cosmesis, and fewer complications [2, 3].

However, there are several limitations to traditional laparoscopy. The learning curve is very long, the hand movements are counterintuitive, and the long instruments working through a fixed entry point cause small movements and even tremors to be accentuated. These factors make fine motor control more difficult. Also, the instruments have a limited range of motion and often require ergonomically challenging positions. This can result in fatigue and frustration by the surgeon during lengthy cases. Lastly, the 2-dimensional optics and the unstable camera platform result in loss of depth perception and difficult visualization, depending on the stability and skill of the assistant operating the camera.

Due to these limitations, many complex surgical procedures are still done as an open procedure. However, with the advent of the da Vinci robotic system, (DRS) developed by Intuitive Surgical, Sunnyvale, CA, USA and approved in April 2005 by the United States Federal Drug Administration (FDA) for gynecologic surgery, many surgeries that would have been done with an abdominal incision are now being performed with minimally invasive techniques utilizing the DRS. 

The term “robot” is derived from the Czech word “robota” coined by the Czech playwright Karel Capek in 1921 in his play *Rossum's Universal Robots*. Since then, robots have become utilized in many industries, most recently the medical field. Initially, the concept of robotics in medicine started with a simple voice-recognition system called HERMES, which controlled the camera, light source, insufflation, and table movements by voice commands. In 1994, the FDA approved AESOP, a single robotic arm that controlled the camera by voice command. In 1999, two arms were added to create ZEUS, which introduced the concept of the surgeon operating at a distance from the patient at a console to control the robotic arms, often referred to as telesurgery. In fact, in 2001, the first telesurgery was done by a surgeon in New York performing a laparoscopic cholecystectomy on a patient in Strasbourg, France [4]. These robotic platforms were initially funded and developed by the Stanford Research Institute, the United States Defense Department, and the National Aeronautics and Space Administration in hopes to bring telesurgery to wounded soldiers in the battlefield. However, due to limitations in telecommunication requirements, its use in the battlefield has not occurred, despite the technological capabilities of the robotic platform. The robotic platform was furthered developed and commercialized by Intuitive Surgical Inc. to create the da Vinci robotic system (DRS). The company then acquired the manufacturers of ZEUS and phased it out of production and the first successful surgery using DRS was performed in Belgium in 1997 [5].

The da Vinci robotic platform consists of three components: the surgeon's console, which directs the movements of the robotic arms, the vision system, and the patient-side cart, which in the latest system has four arms. After placement of port sites and docking the patient side cart, the surgeon sits at the console and is able to view the pelvis through a three-dimensional vision system in high definition. Furthermore, the camera system is stabilized by the robotic platform and easily controlled by the surgeon through foot pedals and arm movements. At the console, the surgeon controls the robotic arms and the EndoWrist instruments with natural hand and wrist motions that mimic movements performed in open surgery. In fact, the EndoWrist instruments are designed with seven degrees of freedom, one more than the human hand. Also, the robotic system is able to reduce tremor and is ergonomic for the surgeon with armrests and adjustable height and eye pieces. It also offers ease of use through foot pedals that control swapping in and out the third robotic arm, moving and focusing the camera, and controlling monopolar and bipolar currents connected to the EndoWrist instruments. All of these components reduce the fatigue, frustration, and strain experienced by laparoscopic surgeons during long or difficult cases. 

Due to such advancements, the robotic platform allows less experienced laparoscopic surgeons to perform more complex procedures. The surgeon is able to progress quickly along the learning curve and accomplish tasks such as intracorporeal suturing and knot tying, ureterolysis, lymphadenectomies, and lysis of dense adhesions with ease and improved visualization. Also, additional assistance can be achieved through the 10–15 mm assistant port to provide suctioning, retraction, vessel coaptation, passage of suture or laparotomy sponges, and even thrombogenic agents as needed. Furthermore, uterine manipulation by an assistant provides another method to improve visualization and access to the pelvis, which is unique to gynecologic surgery. Also, after completion of a total hysterectomy, the vaginal canal offers access to the pelvis for the removal of specimens.

Many gynecologic surgeons now are performing procedures that they never would have been comfortable performing with traditional laparoscopy, affording many patients the option of minimally invasive surgery (MIS). The adoption of the robot has come into play in general gynecology as well as in nearly every subspecialty of gynecology. In general gynecology and reproductive gynecology, the robot is being increasingly used for procedures such as hysterectomies, myomectomies, adnexal surgery, and tubal anastomosis. Also, among urogynecology, the robot has been utilized for sacrocolpopexies and fistula repairs. But perhaps the most profound utilization of the robot has been in the field of gynecologic oncology, where the robot is being increasingly used to perform hysterectomies and lymphadenectomies for endometrial cancer staging, radical hysterectomies and trachelectomies for cervical cancer, and even for the staging and debulking of early ovarian cancer. 

For example, at one major university hospital center, the route of hysterectomy changed significantly after the introduction of robotic surgery. The rate of open hysterectomies declined (52% to 43%), the rate of traditional laparoscopic hysterectomies decreased (18% to 8%) as well as the rate of vaginal hysterectomies (27% to 24%) while the rate of robotic hysterectomies increased from 2.5% to 25% [6]. Also, at another large academic teaching hospital, after the introduction of robotics, the proportion of cases done through minimally invasive surgery increased from 9% to 36% in the third year after introducing the robot [7].

Despite the rapid and widespread adoption of DRS in gynecology, there are no randomized trials comparing its efficacy and safety to other traditional surgical approaches. In fact, the vast majority of the published literature on DRS in gynecology consists of case reports, descriptions of technique, and retrospective case series, and several review articles summarize these publications [8–13]. There was a recent systematic review and meta-analysis comparing the efficacy of DRS to open surgery (OS) and laparoscopy (LSC), but the first publication only included literature related to general surgery [14]. However, a second publication by the same group has recently reported a meta-analysis of observational studies in robotic gynecologic surgery that included studies published up until October, 2009 [15]. Our aim is to update this previously published review. We will focus on comparative observational studies only, understanding the inherent limitations of observational studies due to confounding and bias, and the potential for time-period effects when historical controls are used. Also, there is vast heterogeneity in the studies reported due to differences in institutional practices, surgeons, and their skills as well as their place on the learning curve, and patient populations. Taking these limitations into account, we will objectively and systematically review the current evidence describing the safety and efficacy of DRS in benign and malignant gynecologic disease compared to open surgery (OS) or laparoscopy (LSC). We will evaluate only comparative observational studies in which one type of gynecologic procedure is performed among all subjects being compared. Due to the anticipated heterogeneity between the studies and high likelihood of biased estimates, we will not report overall measures of effect. This note of caution was described in the 2nd of “Systematic Reviews In Health Care: Meta-analysis in context.”

Due to the effects of confounding and bias, such observational studies may produce estimates of associations that deviate from the underlying effect in ways that may systematically differ from chance. Combining a set of epidemiological studies will thus often provide spuriously precise, but biased, estimates of associations. The thorough consideration of heterogeneity between observational study results, in particular of possible sources of confounding and bias, will generally provide more insights than the mechanistic calculation of an overall measure of effect [16].

Nonetheless, we predict that DRS will have equal outcomes compared to LSC and improved outcomes compared to OS, with no increased safety concerns across the majority of gynecologic procedures. We anticipate a paucity of data concerning long-term outcomes, limiting our ability to make recommendations regarding the utility of DRS in regards to particular gynecologic conditions.

## 2. Methods

On June 8th, 2011, relevant articles were identified through a Medline/pubmed search using the keywords from Title/Abstract fields: Robot* OR Davinci OR da Vinci AND either Gynecolog* OR Hysterectomy OR Myomectomy OR tubal OR adnexal OR ovar* OR sacrocolpopexy OR Endometrial cancer OR Uterine cancer OR cervical cancer OR cervix OR ovarian cancer, from 1990 to June 8th, 2011. The search was limited to English language and published studies only. A manual inspection was also made of the references cited in the articles found. Articles were identified by their title, and, among all relevant articles, the abstracts were reviewed and full text when necessary.

 We included studies that reported on perioperative outcomes of a group that underwent DRS compared to either historical or concurrent controls, matched or unmatched, that had similar procedures performed but underwent LSC and/or OS. Primary outcome data abstracted includes perioperative data such as operating time, blood loss, lymph-node count when applicable, and length of hospital stay as well as rate of blood transfusion, any intraoperative or postoperative complications, and conversions to open surgery. When short- or long-term clinical outcome data was reported, it was also collected. Demographic data such as age, body mass index (BMI), surgical indications, and uterine weight were also abstracted when appropriate.

We excluded studies if the subjects were nonhuman, if there was no control group, if the groups being compared appeared significantly different from one another, if the total number of cases in the DRS group was less than 20 to minimize potential learning curve biases, or if the study methodology appeared flawed or was not well described. Also, quality of the studies were assessed, and if we felt they did not clearly define the question, lacked appropriate followup of the patients, or had dramatic inequalities in patient management between the groups, the study was excluded.

Meta-analysis of observation studies in Epidemiology reporting guidelines was followed when conducting this study [17]. Information abstracted from each identified study was tabulated in a collection form for subsequent reference and analysis.

## 3. Results

A total of 665 studies were identified, of which 434 were found to be unique. From these 434 studies, 45 met inclusion criteria and upon further review 13 of these studies met exclusion criteria. One of these studies that met exclusion criteria (*n* < 20) was still included due to its superior design and novel findings [18]. The remaining 12 studies were excluded for sample size less than 20 (10), a variety of procedures among groups (1), and questionable balance between groups as well as methodology of study (1). The remaining 33 studies were then included in this systematic review, and their outcomes are summarized in tabulated form.

## 4. Benign and Reproductive Gynecologic Surgery

### 4.1. Tubal Anastomosis

The first reports of the da Vinci robot were in urologic procedures in 1995 and cardiac procedures in 2001 [19, 20]. In 2000, the first case of robotic surgery in gynecology was reported, a tubal anastomosis (TA) [21]. To date, two studies describe robotic compared to other approaches in performing TA [22, 23]. Unfortunately, both these studies meet exclusion criteria (*n* < 20), but, due to the paucity of data and novelty of their data, we will discuss them for informative purposes. The first study conducted by Goldberg and Falcone at the Cleveland clinic includes the first ever performed case in addition to their subsequent 9 patients that underwent TA robotically with ZEUS compared to their subsequent 15 patients that underwent LSC TA. Due to the small number of subjects (*n* = 10 in the robotic group) and noncomparability of groups (women in the LSC group were significantly older and tended towards having larger BMI and prior surgeries), this study has many potential biases. In addition, 6 of the 15 patients in the LSC group were lost to followup. Also, the robotic group excluded patients with other infertility factors, but the LSC group included 8 patients with other infertility factors. Nonetheless, they reported higher tubal patency rates (90% versus 69%) and pregnancy rates (50% versus 38%)—after adjusting for loss of followup and excluding those with other infertility factors—in the robotic group compared to the LSC group, but these differences were nonsignificant. There were significantly longer operating room times and estimated blood loss (EBL) in the robotic group of 124 minutes and 50 mL, respectively, compared to LSC TA.

The second study evaluated 18 patients that underwent DRS for TA compared to 10 historic controls (HC) who underwent OS for TA one year prior [23]. The same surgeon performed every one of these cases, and the investigators excluded other infertility factors between both groups. They measured operative times, hospitalization time, charges, complications, postoperative recovery, postoperative tubal patency, and clinical outcomes. Demographic data as well as mean time from sterilization were comparable between the groups. Statistically significant differences included longer operative times in DRS versus OS of 201 min versus 155 min; however, the console time was only 156 min, comparable to the operative times in the OS group. Patients in the DRS group were all discharged within 4 hours postoperatively; whereas, in the OS group the average length of hospital stay postoperatively was 35 hours. There was only one complication, a trocar injury to the inferior epigastric artery in the DRS group, which was recognized and cauterized intraoperatively. There were no conversions to OS. Also, analgesia use was less, and time to return to activities of daily living (ADL) was faster in the DRS group (11 versus 28 days). Clinical outcomes were not different although the followup was shorter for patients in the DRS group versus OS group. Lastly, they found that DRS TA was cost effective in terms of cost per live birth as compared to OS. Increased cost was balanced by increased recovery times and hospital stays in the OS group [23]. 

#### 4.1.1. Recommendations: Level 2− Grade D

Although these studies both have potential for bias, they do suggest that even at the beginning of their learning curve, DRS for TA does appear to have advantages in terms of faster recoveries over OS and at least equal outcomes compared to LSC, but further studies and randomized trials are warranted to compare efficacy and safety of DRS in TA.

### 4.2. Adnexal Surgery and Endometriosis Treatment

Most of the literature concerning adnexal surgery is not well characterized as many of the reported adnexal procedures are done concurrently with hysterectomies. Often, isolated oophorectomies or cystectomies are easily accomplished with traditional laparoscopy as initially described in 1979 [34]. However, cases in which adhesive disease, advanced endometriosis, or large-complex masses are present, the use of robotics may make completion of the desired procedure more feasible without necessitating converting to OS. Nezhat et al. reported, in 1999, the difference in operative times for hysterectomies with (*n* = 17) and without BSO (*n* = 10) equaling an additional 99 minutes [35]. Also, in an earlier series by the same group, they described utilizing the robot to treat endometriosis in two patients, which entailed resection of endometriotic lesions, lysis of adhesions, ovarian cystectomy, and repair of the ovary after cystectomy. Neither patient required a conversion to open or experienced intraoperative or postoperative complications [36]. Liu et al. also reported a successful case of a partial bladder resection due to infiltrating endometriosis using DRS [37]. At the same time, Chammas et al. also reported a case of endometriosis managed with DRS that included surgical resection of a bladder mass and rectal nodule as well as excision of an ovarian cysts and resection of peritoneal endometriotic implants [38]. 

The initial report of the feasibility and efficacy of LSC adnexectomy over OS was completed in 1994 by Pittaway et al. [39]. This report was a retrospective case-control study using historic controls (HCs). They reported that LSC had improved operative times, blood loss, hospital stay, recovery time, and costs, with no difference in complications or clinical outcomes. Most adnexal surgery is now done laparoscopically. Since the introduction of DRS, the only study comparing outcomes of LSC to DRS in adnexectomy was published in 2009 by Magrina et al. [24]. They evaluated 85 patients that underwent DRS adnexectomy compared to 91 patients that had LSC adnexectomy during the same time period, concurrent controls (CCs), and by the same surgeons (SSs). The only difference between the groups was that women in the DRS group had higher BMIs and higher anesthetic risk class. This difference may account for the increased median OR time in the DRS group (77 versus 62). However, among patient with BMI over 30, there was no difference in median OR time in the DRS group (66 versus 58). Also, although overall blood loss was not different, among a subset of women with BMI over 30 DRS had less blood loss compared to LSC. Otherwise complication rates and hospital stays were equivalent with no blood transfusions or conversions (Table 1).

#### 4.2.1. Recommendation: Level 2+ Grade D

The advantage of DRS for adnexectomy likely lies among a subgroup of obese women where a difficult dissection is anticipated.

### 4.3. Myomectomy

Myomectomy remains the gold standard for surgical treatment of women with symptomatic myomas that desire future fertility. While many adnexal surgeries can be performed with traditional laparoscopy, myomectomies are often more technically challenging, and the vast majority are still performed by OS despite the introduction of laparoscopic myomectomies by Semm in 1979 [34] and the benefits of OS substantiated by a prospective randomized controlled trial in 2000 with pregnancy as the primary outcome [40]. The underutilization of LSC myomectomy is likely due to the extensive suturing of the myoma bed and uterine serosa as well as the precise dissection and torque required to remove the myoma. Additionally, the risk of uterine rupture during pregnancy after LSC myomectomy has been reported as 1 percent. Due to such risk, only skilled LSC surgeons should perform this procedure. However, robotic surgery, with its ease of suturing, knot tying, and improved visualization, affords the option of a minimally invasive approach to myomectomies among less advanced laparoscopic surgeons. 

There have been several observational studies [35, 36, 41, 42] and case reports [43, 44] describing the feasibility, complications, and success of DRS myomectomies. Among theses studies, only the case series of 35 patients reported conversions to OS (2/35) [41]. To date, there have been five comparative observational studies of DRS for myomectomy (Table 1).

The first study was published by Advincula et al. in 2007 [25]. They performed a retrospective case-matched study on a total of 58 patients that had DRS or OS for myomectomy in 2000 to 2004. One surgeon performed all the DRS cases and six different surgeons performed the OS cases. They were matched on myoma weight, age, and BMI. They found that the DRS had less EBL, a shorter length of hospital stay, and fewer complications, but longer operating times and larger hospital costs with no difference in professional reimbursement. Recently, Ascher-Walsh and Capes have compared 75 cases of DRS myomectomy to 50 HCs of OS myomectomy [27]. They only included patients with 3 or less myomas and uterine size 20 weeks or less. The groups were comparable and they found longer operative times for DRS but less EBL, shorter hospital stays, and a faster return to normal diet. There was also significantly less febrile morbidity with DRS compared to OS. They also concluded that DRS for myomectomy is superior. 

The first study of DRS compared to LSC for myomectomies was done by Nezhat et al. in 2009 [18]. They compared 15 DRS to 35 LSC myomectomies. All cases were performed by the same surgeon (C.N.) and during the same time period. The patients were matched by age, BMI, parity, previous abdominal surgeries, size, number, and location of myoma. Although this study has *n* < 20 for the DRS group, due to its excellent design (matched concurrent controls with the same surgeon), we included their results in our systematic review. They reported increased operative times in the DRS group but otherwise equal EBL, hospital stay, and pregnancy outcomes. However, the additional cost (hospital charge) of DRS versus LSC was $21,500. They therefore concluded that there are no significant advantages of DRS compared to LSC and that the role of the DRS may be better reserved for surgeons at the start of their learning curve. Bedient et al. in 2009 performed a comparable study with similar short-term outcomes after adjusting for differences in uterine size, number of fibroids, and size of largest fibroid using a multivariate regression model [26]. Despite the equivalence of their results, they did conjecture that robotic surgery improved fibroid enucleation, layered closure and also lowered the number of uterine incisions potentially protecting against the risk of uterine rupture in subsequent pregnancies. They also noted that important long-term outcomes were not assessable due to their short followup, similar to the majority of the other studies.

The largest and most recent study to date by Barakat et al., compared DRS to LSC and OS [45]. Historic controls were used in this study over an extended time period from 1998 to 2008. There was no mention of the surgeons who performed the surgeries or where they were performed. Also, the groups were fairly comparable, except that in the LSC group patients had smaller and lighter myomas and patients in the OS group had higher BMIs and more previous LSC surgeries. In this study, LSC was more often used for patients with smaller myomas, while DRS allowed performance of myomectomy in patients similar to those in the OS group. Nonetheless, they found longer operative times in the DRS group compared to OS but also shorter hospital stays and fewer complications. Similarly to previous studies, DRS was comparable to LSC in terms of blood loss, operative time, and hospital stay. However, there were more complications (two blood transfusions and one bowel injury) in the LSC group compared to no complications in the DRS group. Yet, due to the historic nature of the controls and potential difference in surgeons, it is difficult to attribute these differences in complications to the use of LSC over DRS alone. However, LSC and DRS had a significantly decreased rate of blood transfusions compared to DRS [45]. 

#### 4.3.1. Recommendations: Level 2+ Grade C

All of these studies suggest that DRS is equivalent to LSC myomectomy, but likely only in the hands of experienced LSC surgeons. However, DRS may be superior to LSC for patients with large or multiple myomas who traditionally would not be considered candidates for MIS. Therefore, either among less experienced laparoscopic surgeons or among patients predicted to have difficult myomectomies DRS may be preferable over LSC. On the other hand, DRS appears to be superior to OS although no studies to date have looked at long-term outcomes. The risk of uterine rupture could theoretically be increased with DRS compared to OS. Therefore, until long-term data are available we hesitate to advise routine DRS myomectomies over OS. 

### 4.4. Hysterectomy

Hysterectomies are the most common nonpregnancy-related procedure among women with over 600,000 performed annually in the United States, and 90% are for benign conditions [46, 47]. In the past, the only two options for performing a hysterectomy were an abdominal approach or a vaginal approach. However, in 1989, laparoscopy was first used to perform a hysterectomy [48]. It wasn't until over a decade later, in 2002, that the use of the da Vinci robot for hysterectomies was first reported [49]. The investigators conducted a retrospective case review of 11 patients with a variety of indications for simple hysterectomy including benign and malignant processes, with one patient undergoing a staging procedure for ovarian cancer. Their operating times varied from 270 to 600 min. with an average EBL of 300 mL (range, 50 to 1500 mL). The average hospital stay was 2 days (range, 1–3 days). There were no conversions to open surgery; however, one patient experienced bleeding from an infundibulopelvic ligament requiring minilaparatomy and one unit of blood transfusion. Then, Marchal et al. in 2005 reported on 30 cases done in Belgium and France for malignant and benign diseases [50]. They reported shorter operating times of 166 min. for benign cases and 181 min. for malignant cases with an average EBL of 83 mL. They did not have any intraoperative complications, but one conversion to open due to body habitus and a 17% postoperative complication rate. Beste et al. in 2005 reported a case series of 10 patients undergoing hysterectomy for benign conditions and showed operative times of 148 min to 277 min with an EBL of 25 to 350 mL and hospital stay averaging one day. Initially, they intended to treat 11 patients with a robotic approach, but one was converted to an open procedure in order to control bleeding during skeletonizing of the uterine vessels. Also, one patient had a cystotomy, but otherwise no other complications. Many other similar retrospective case series have reported a variety of different outcomes and complication rates [35, 42, 51–54].

Lenihan et al. in 2008 prospectively evaluated 113 consecutive gynecologic cases performed robotically, 91 of which were hysterectomies for benign conditions [55]. Two surgeons performed all the cases after undergoing computer-based and porcine laboratory training, and their learning curves were assessed. Robotic console and operative times plateaued after approximately 50 cases at 50 min. for console time and 90 min. for total operative time. While total operating times and EBL were affected by uterine weight, robot console time was not significantly affected by uterine weight. However, the time increased on average only 10 min. among cases with larger uteri, which was due to morcellation and removal of the uterus. Also, the EBL decreased from 160 mL in the first 25 cases to only 50 mL in cases 76–100. There were no conversions to open, and only twice did they convert to a laparoscopic assisted vaginal hysterectomy (LAVH) to facilitate removal of the uterus. They did report 4 complications including a delayed ureteral injury near the cuff, a 6-week cuff dehiscence, a post-operative fever, and a vaginal laceration from the removal of the uterus [55].

Bell et al. in 2009 also reported on the learning curve among 100 robotic hysterectomies performed by a single surgeon [56]. They collected operative times and complications retrospectively and found that the total operating time declined as case number increased with the times in the first, second, third, fourth, and fifth quintiles being 124, 94, 85, 88, and 81 minutes. Also, the rates of complications were highest in the first 20 cases (15%), but only 5% for the remaining cases. Therefore, they concluded that the maximum improvement in surgical times occurred after only 20 cases [56], contrary to 50 cases as reported by Lenihan et al. [55]. Despite the short learning curves, the vast majority of minimally invasive hysterectomies in the United States, 95%, are being performed LSC without robotic assistance [57].

While there are many studies as mentioned above which are reporting outcomes of DRS for hysterectomies in benign disease, there are only 4 studies to date reporting comparative observational data of DRS versus LSC hysterectomy. No studies compare DRS to OS for hysterectomy. Payne and Dauterive reported the first study in 2008. They retrospectively reviewed 200 cases of consecutive hysterectomies completed before and after the implementation of a robotics program at their institution with the first 100 cases intended to be performed by LSC and the second 100 cases intended to be performed by DRS [29]. All the hysterectomies were done for benign conditions, and the mean uterine weights and body mass index (BMI) did not differ between the two groups. The overall operative time was longer in the LSC group (120 versus 92 min.), but, when comparing the operative times of the last 25 DRS cases to the LSC cases, the DRS cases were significantly shorter (79 versus 92 min.). Also, the average EBL of the DRS cases were half that of the laparoscopic cases (61 versus 113 mL) and the patients had shorter hospital stays (1 versus 1.6 days). Twenty-percent of the intended LSC cases were done as an exploratory laparotomy or converted to OS (eleven for large uterine size, 8 due to adhesions, and one case due to a tuboovarian abscess) while only 4% of the DRS cases were converted to OS (one due to a cystotomy, and 3 due to large uterine size). There was one enterotomy and one vaginal tear in the LSC group and one cystotomy and one cuff infection in the DRS group. Thus, the robotic group had improved outcomes with no difference in complication rates [29].

Although not a comparative study, Payne et al. have recently reported on a multicenter retrospective case series of DRS hysterectomies performed on uteri of at least 250 g. [58]. The reported only 4 conversions to OS among 256 cases (1.6%) with a minor complication rate of 1.6% and a major complication rate of 2.0%. Interestingly, three of the four patients with conversion to OS had uterine weights above 1,000 g. Uterine weight above 500 g was independently associated with increased EBL and operative times in multivariable linear regression models with approximately 40 additional minutes of operative time and 60 mL increased blood loss [58]. This study highlights the utility and safety of robotic hysterectomies for large uteri; however, careful consideration should be made for those with extremely large uteri.

Shashoua et al. in 2009 compared 24 patients that underwent DRS hysterectomy versus 44 patients that underwent LSC hysterectomy [30]. Of note, one patient in each group underwent a simple hysterectomy due to a malignant process. They found that DRS was associated with a significantly shorter hospital stay, a decrease in narcotic use postoperatively, but a longer operative time compared to LSC. However, there was no difference in EBL or drop in hemoglobin. Although DRS had an increased operative time, patients in the DRS group tended to have larger uteri, and a significantly larger proportion underwent morcellation (23% versus 2%). In fact, in a multivariate analysis, only need for morcellation, uterine weight, and BMI were independently associated with increased operative times, and not utilization of DRS. Also, there were no conversions to OS in either group and the rates of complications were comparable.

Sarlos et al. in Switzerland have recently published a prospective observational study of 40 patients undergoing DRS hysterectomy with uteri <500 g. compared to 150 matched (surgeon, age, BMI, uterine size) HCs [31]. They found that DRS had significantly longer operative times, but no difference in blood loss, hospital stay, or complication rates. They also evaluated costs of the two procedures and found DRS cost 1,916 more Euros than LSC hysterectomy. Therefore, they concluded that while DRS is a feasible and an interesting new technique with comparable outcomes to LSC, they hesitated to promote its adoption for benign hysterectomies due to concerns of cost until randomized trials were conducted to clearly elucidate its potential benefits.

 Giep et al. compared 237 DRS hysterectomies performed by 2 surgeons in a community hospital to 265 LSC-assisted vaginal hysterectomies (LAVHs) performed by the same 2 surgeons and 9 of their partners [32]. Although the DRS group had more complexity (prior abdominal surgeries and more procedures for endometriosis and pelvic reconstruction) as well as greater uterine weights, DRS still had shorter operative times, EBL, and length of hospital stay. They also reported similar complication and conversion rates. However, they were only able to assess complications postoperatively after discharge in the DRS group, not in the LAVH group. This reporting bias favors the LAVH group, making the final statement of equivalence in complication rates presumptively stronger in favor of DRS. Furthermore, through multivariate regression analysis, only LAVH, obesity, older age, and uterine weight >250 g were independently associated with longer operative times. They conclude that DRS is advantageous over LSC hysterectomy, specifically LAVH.

Another study by Pasic et al. utilized the Premier hospital database, which contains complete patient billing, hospital costs, and coding histories of more than 600 health care centers in the U.S [57]. They identified 36,188 patients that had MIS hysterectomies, with slightly less than 5% of these patients undergoing DRS and only 13% of the 358 hospitals identified had access to the DRS. While they did obtain information on patients' comorbid conditions, there was no mention of the patients' uterine weights, BMI, or history of prior surgeries. Also, there were a large variety of procedures performed including radical hysterectomies for malignancies, lymphadenectomies, supracervical hysterectomies, and the vast majority of the procedures were LAVH. They did control for surgery types, indications for surgery, region, hospital type, comorbid illnesses, and age in multivariate regression analysis to assess the effects of robotic or no robotic assistance on hospital costs, and surgery time, and length of stay. However, due to the heterogeneity of their patients, procedures, and surgeons, and because identification of complications could only be completed through reported ICD-9 codes, this study was excluded. Nonetheless, they found similar adverse events across DRS and LSC hysterectomies, but significantly longer surgical times with DRS and shorter lengths of stay. However, due to higher costs of DRS, they cautioned against its routine use in LSC hysterectomies [57].

#### 4.4.1. Recommendations: Level 2+ Grade C

DRS may offer improved recovery, less blood loss, and decreased conversion rates to OS compared to LSC. DRS may take longer than LSC hysterectomies, but this most likely occurs at the onset of the learning curve and is most likely attributable to larger uterine weights and patient BMIs, rather than surgical approach. Therefore, DRS for simple hysterectomies for benign design is most likely the preferred modality for less experienced laparoscopic surgeon or for patients with larger uterine size or anticipated difficult surgeries that would require converting to OS. 

## 5. Urogynecologic Surgery

### 5.1. Sacrocolpopexy

Approximately 200,000 women in the US annually undergo surgery for vaginal vault prolapse, and 30% of these women will require reoperation for recurrent prolapsed [59, 60]. Abdominal sacrocolpopexy has emerged as the gold standard treatment for recurrent prolapse with a 90% success rate at 5 years [61]. While recent studies have shown comparable outcomes with LSC sacrocolpopexy [2], due to the limitations of LSC and the suturing required during sacrocolpopexy, many less experienced LSC surgeons have begun utilizing the DRS. The first case series of DRS sacrocolpopexy involved 5 patients at Mayo Clinic in the Department of Urology, and they later included another 15 patients in a subsequent publication [62, 63]. They also subsequently reported long-term outcomes on theses patients with an average followup of 24 months and among 21 patients, there were one recurrent rectocele, one recurrent vault prolapse, and two mesh erosions (thought to be due to overly aggressive bladder dissections done in earlier cases) [64]. The Cleveland Clinic also reported a case series of 15 patients undergoing DRS sacrocolpopexy or hysteroscacrocolpopexy and reported mean operative times of 317 minutes, EBL of 81 mL, and an average hospital stay of 2.4 days. They also reported three conversions: to LSC, vaginal, or OS due to bowel adhesions. They only had one complication, a bowel serosa tear, and they reported mean preoperative and postoperative POP-Q stages of 3.1 and 0. Although they concluded that DRS offered a shorter learning curve than LSC and provided durability equal to OS with decreased morbidity, they did note that the loss of haptic feedback with DRS made placement of vaginal and sacral sutures more difficult [65].

To date, there are only two reports of observational data comparing DRS, LSC, and OS in performing sacrocolpopexies [33, 66]. The later report comparing DRS to LSC was excluded due to less than 20 DRS surgeries in the treatment group. Nonetheless, they did report longer operative times in the DRS group compared to LSC sacrocolpopexies and shorter hospital stays among DRS with equivalent rates of complications. They also reported clinical outcomes at an average of 29 months followup and found similar objective cure rates of 93% and 91% with 1 recurrent prolapse (≥stage II) in the DRS group versus 2 recurrent prolapses (≥stage II) in the LSC group. Also, there were no mesh exposures or erosions [66]. The first report, however, by Geller et al. in 2008 meets all the inclusion criteria [33]. It is a retrospective review of 73 DRS sacrocolpopexies performed by one surgeon versus 105 OS sacrocolpopexies performed by another surgeon at the same institution. Although the patients were similar in age, race, and BMI, patients in the DRS group had more significant prolapse based on their preoperative POP-Q scores and were also more likely to undergo hysterectomy and less likely to undergo other concomitant procedures for prolapse. Among the DRS group, they reported longer operative times, less EBL, and shorter hospital stays. Due to the differences in concomitant procedures, they performed multivariate adjusted analyses to control hysterectomy, concomitant procedures, as well as lysis of adhesions and BMI and still found significantly longer operative times and less EBL in the DRS group. They had one conversion to OS due to extensive adhesions and a cystotomy in each group that was repaired intraoperatively without complications or conversions. There was one pulmonary embolism in the DRS group, but otherwise rates of complications were equivalent. They also reported equal 6-month post-operative POP-Q points between the DRS and OS groups, except for a significant improvement in the C point POP-Q among patients that underwent DRS (*P* = 0.008). This figure is impressive considering that patients in the DRS group had significantly worse C point scores preoperatively compared to patients that underwent OS. Their results are encouraging for DRS, but they should be cautioned because of their lack of long-term followup [33].

The DRS is also used for other urogynecologic procedures such as fistula repairs, but only case reports [67] and case series have been reported to date [68–70]. All of these reports show success with DRS and no complications. There has also been a case report of DRS sigmoid vaginoplasty in a 17 year-old 46 XY adolescent that took 9 hours and 45 minutes to complete with no complications and successful dilation at 10-month followup [71]. Thus, DRS appears to have wide applications in urogynecology, but the literature supporting these modalities is still in its infancy, and costs effectiveness needs to be continuously assessed.

### 5.2. Recommendations: Level 2− Grade D

Although the literature would suggest at least an equivalence of DRS to other surgical approaches, due to the increased cost of DRS, we will need further information from randomized trials to better characterize the utility of DRS before adopting it as routine use for sacrocolpopexies. Also, as mentioned previously, long-term clinical outcomes are lacking. The results of a randomized controlled trial just completed at the Cleveland Clinic comparing DRS and LSC in sacrocolpopexy will be instrumental in helping discern the utility of DRS in urogynecology.

## 6. Gynecologic Oncology Surgery

### 6.1. Uterine Cancer

Uterine cancer is the most common gynecologic cancer among women in the US, accounting for 6% of all female cancers, and approximately 2.6% of women will be affected by uterine cancer in their lifetime [72]. The primary management of uterine cancer involves a hysterectomy, bilateral salpingooophorectomy (BSO), and when feasible pelvic and para-aortic lymphadenectomy to better characterize the disease due to the risks of occult spread. While the utility of lymphadenectomy and complete staging during surgery is still debated, many gynecologic oncologists use information from staging to help tailor adjuvant therapies [73]. Since Childers first demonstrated the feasibility of LSC in staging uterine cancer, there has been an increase in the use of LSC in staging women with uterine cancer [74]. This change has resulted in decreased morbidity postoperatively. In fact, a recent randomized controlled trial from the Gynecologic Oncology Group, the LAP2 trial, has compared LSC to OS for surgical staging of uterine cancer and found similar intraoperative complication rates, but fewer postoperative complications and shorter hospital stays, with no difference in detection of advanced stage disease [75]. They also showed a modest improvement in quality of life [76]. Unfortunately, only 6-week postoperative outcomes were assessed, so no conclusions regarding differences in long-term outcomes can be made. However, they reported a 26% conversion rate to OS among the patients randomized to LSC, mostly due to poor visibility [75]. With the DRS, the challenges of visibility with LSC can be overcome as well as many other limitation of LSC. Hence, the field of gynecologic oncology has begun adopting the DRS in performing surgical staging of uterine cancer due to its shorter learning curve and ease of use. In fact, a recent survey of gynecologic oncologist has found that nearly 40% of respondents felt that robotic surgical training was required as a part of their career goals, and 73% had performed a robotic hysterectomy [77]. Also, at a large academic teaching hospital, after the introduction of the DRS, the proportion of cases done minimally invasive for uterine cancer staging increased from 6.4% prerobotics to 80.5% in the third year after its introduction [7].

Many studies looking at DRS in uterine cancer staging include case reports and case series. However, more recent studies have begun to look at comparative observational data of DRS versus OS and LSC in uterine cancer staging; in fact, fourteen studies have been published to date. Unfortunately two of these studies had sample sizes less than 20 and were excluded [78, 79]. Of the twelve included, the size of the robotic group ranged from 25 to 377, which, not surprisingly, included the first and the most recent study reported to date and were from the same authors at the Swedish Medical Center in Seattle, WA, USA (Table 2). Also, among the selected studies that compared DRS to OS, operative times were significantly longer for DRS except for one study with a nonsignificant difference [80], but all studies showed a significant improvement in blood loss and duration of hospital stay among DRS compared to OS (Table 2). The lymph node yields were also nonsignificantly different between DRS and OS, except for one study with greater nodes in the DRS group [81] and one study with fewer nodes in the DRS group [82]. In regards to complications, DRS had either fewer intraoperative and postoperative complications or no significant difference compared to OS. The important difference was that OS tended to have more wound complications whereas DRS tended to have more cuff complications. 

The differences between DRS and LSC are not as drastic or consistent. For example, operative times were either nonsignificantly different or shorter for DRS compared to LSC (Table 2). However, one study reported shorter operative times for LSC compared to DRS, and this was in a study where only one surgeon performed all the procedures and was very experienced with LSC [90]. Five studies reported significantly less blood loss with DRS compared to LSC, whereas two studies showed no significant difference. Half the studies showed a decreased duration of hospital stay with DRS compared to LSC, whereas the other half showed no significant difference. In regards to node yield, three studies showed a benefit with DRS, four studies showed no difference, and one study showed a benefit with LSC. Complication rates were generally equivalent between DRS and LSC, except for 2 studies that showed worse post-operative complication rates with LSC, and one of these demonstrated worse intraoperative complications for LSC as well. However, in that study all the cases were done by a single surgeon who had just finished fellowship and all the LSC complications occurred within his first 40 cases [82].

Due to the observational nature of these studies, and the aforementioned studies in general and reproductive gynecology, many are fraught with potential biases including selection bias, investigator bias, recall bias, and many random effects due to the uncontrolled nature of the studies. For example, many of the studies report some differences in demographics between the comparison groups. Also, the data collection methods (e.g., retrospective and prospective) were often different for the groups due to the use of HCs, which can cause time period effects. For example, traditional practice 5 to 10 years ago likely kept patient in the hospital longer after surgery compared to current practice. Also, studies including different surgeons with undoubtedly different skills and experience will change outcomes between the groups. Even when subjects are matched by surgeon or a study includes only one surgeon, outcomes can be very different across studies. Also, the surgeons performing the procedures within and between studies are most likely at different time points in their learning curve causing additional variability in outcome data. Thus, outcomes are better assessed in the context of the studies' methodology, rather than as a composite score.

The first study, by Veljovich et al. published a heterogeneous study of their first 113 robotic cases and identified 25 that were done for uterine cancer staging and included hysterectomy and lymph-node dissection which was compared to 131 historic and concurrent controls of OS for uterine cancer staging [83]. They found longer operative times in DRS but shorter hospital stays and less EBL with comparable node yields. There was a trend towards higher major complications in OS (21% versus 8%). There were 9 wound complications in the OS group compared to none in the DRS group. There was one case of a cuff dehiscence that required reoperation in the DRS group, and none in the OS group. There was one reported conversion to OS secondary to a BMI over 49. They also compared DRS to 4 cases of LSC, which had equivalent short-term and perioperative outcomes, but, due to small case numbers, no conclusions can be made [83].

Boggess et al. then published their report of 103 DRS for uterine cancer staging compared to 81 LSC-staging and 138 OS-staging procedures [81]. They found that although patients in the DRS group had a higher average BMI, EBL was less, hospital stay was shorter, and node yield was higher as compared to OS and LSC. Also, postoperative complications were significantly less among the DRS group compared to the OS group. However, complication rates and conversion rates for LSC and DRS were equivalent. They concluded that DRS was superior to OS for uterine cancer staging and may even be preferable over LSC. From the same institution, Gehrig et al. published a similar study looking at a subset of women with BMI over 30 who were undergoing DRS or LSC for uterine cancer staging [84]. In this subset of women, DRS was associated with significantly less operative times, EBL, and hospital stay, as well as increased node yield. Complication rates were equivalent and there was one conversion to OS from LSC in a morbidly obese woman. Their results suggest that DRS is superior to LSC for obese and morbidly obese women undergoing surgical staging of uterine cancer.

Bell et al. in 2008 published another study comparing all three modalities (DRS, OS, and LSC) for uterine cancer staging, but in a private practice with all the cases being done by the same surgeon [86]. They also performed a cost analysis of the three modalities using the outcomes they reported. From a total of 110 cases, they found that DRS had longer operative times compared to OS, but operative times were equivalent to LSC. DRS, however, had less EBL than OS, and fewer complications than both OS and LSC. Hospital stay was shorter for DRS compared to OS, but not different from LSC. When evaluating the time to return to normal activity, DRS was significantly less than OS and LSC (24 versus 52 versus 32 days, resp.). Additionally, there was no difference in node yield between the three modalities. In their costs analysis, they assessed costs rather than hospital charges, which, as they mentioned, is a more accurate reflection of the financial impact of the modalities on the health care system. After accounting for costs of hospitalizations, the direct and indirect costs of OS were on average over $4500.00 more than DRS. However, DRS was on average $642.00 more expensive than LSC. They concluded that although DRS uterine cancer staging was longer, it was preferable over OS and LSC [86].

DeNardis et al. then looked at 56 patients that were scheduled to undergo DRS for uterine cancer staging by several different surgeons during the first 14 months of starting their DRS at the Florida Hospital Cancer Institute [87]. They were compared to 106 consecutive patients that underwent OS immediately before starting their DRS program. However, patients that had DRS were younger, thinner, and tended to have less cardiopulmonary disease. Still, they found that DRS had longer operative times, but less EBL, blood transfusions, duration of hospital stay, with no difference in node yield. Also, major perioperative complications were less with DRS versus OS (3.6% versus 20.8%). They reported a conversion rate of 5.4% due to one case of minor bleeding associated with poor visualization and the other case was secondary to a posterior fibroid obliterating the cul-de-sac.

Seamon et al. at Ohio University compared 105 DRS cases performed by two experienced laparoscopic surgeons to those surgeons' previous LSC cases in the 7 years prior [88]. Although patients in the DRS group had higher BMI, they still had improved EBL, duration of hospital stay, and equivalent node yield, but, as expected, higher operative times compared to LSC. They also noted fewer blood transfusions with DRS, but complication rates were otherwise equal. Conversion rates were also less for DRS versus LSC (12% versus 26%). The same group, in combination with the University of Alabama, looked at 109 DRS cases compared to 191 OS cases for women with uterine cancer undergoing staging procedures with a BMI of 30 or greater [89]. Cases were matched by BMI and surgeon, but the DRS patients were still younger with fewer prior abdominal surgeries; however, they had more comorbid illnesses. Also, the final analyses were as treated (e.g., not including DRS cases that were converted to OS). Ninety-two of the 109 (86%) DRS cases were completed with DRS and compared to their 162 matched OS cases. They found longer operative times for DRS versus OS, but as expected improved EBL, duration of hospital stay, blood transfusion rate, complication rates, and wound complications. Yet, there was no difference in node yield.

Jung et al. in Korea looked at 28 cases of DRS, 25 cases of LSC, and 56 cases of OS for uterine cancer staging, all data collection was prospective, and two surgeons performed all the cases [80]. Unlike the techniques described in the majority of US studies, they only used 3 robotic arms and they performed a LSC tubal ligation prior to placement of the RUMI uterine manipulator. Also, their patient population was much thinner with an average BMI of approximately 24. However, the groups were equally balanced across all demographic variables and uterine size. They reported longer hospital stays in the OS group and increased blood transfusions and complication rates as compared to both DRS and LSC. However, there were no differences in operative times. Node yield was less in LSC compared to DRS, but equivalent between DRS and OS. They also reported no conversions to OS in the DRS or LSC groups.

Cardenas-Goicoechea et al. at the University of Pennsylvania compared 102 DRS cases and 173 LSC cases of uterine cancer staging by a single experienced laparoscopic surgeon [90]. Data was collected retrospectively, and the LSC cases were done in the 4 years prior to the DRS cases. Both groups were similar across demographics and uterine size. Interestingly, they found longer operative times in the DRS group, but less blood loss compared to LSC. There was otherwise no difference in length of stay, node yield, and rates of intraoperative and postoperative complications. However, they noted lower rates of ureteral complications with DRS due to the improved visualization allowing for easier identification and manipulation of the ureters. 

Lim et al. at the Center of Hope in Reno, Nevada evaluated the outcomes between the three different surgical approaches at the onset of learning among a single surgeon in a solo practice [91]. His first 56 DRS cases were compared to his initial cases of LSC (*n* = 56) and OS (*n* = 36) cases for uterine cancer staging. Patients in the DRS group were thinner, but otherwise the groups were well balanced. They found that DRS cases on average took longer than OS, but were faster than LSC. Blood loss and hospital stay was improved in the DRS group compared to both OS and LSC. However, node yield was significantly higher in OS and LSC compared to DRS. Although there were more conversions to OS among LSC cases, this number was not significantly different. However, there were significantly more intraoperative complications among LSC cases. This correlation is likely due to the steep learning curve of LSC. In fact, the learning curve for DRS began to decrease in slope after to 20th case and then plateaued, whereas for LSC the operative times continued to decline even after the 40th case and never reached a plateau in this study. The same group then published their continued experience with DRS compared to only LSC, which included 122 DRS cases and 122 LSC cases (including cases reported in the previous study) [82]. These groups were similar across age and BMI. Using a regression model, they determined that the learning curve was reached after the 24th DRS case and the 49th LSC case. However, they noted that learning curve for LSC, even after the 49th case, continued to appear steep and did not plateau. Similar to their prior study, DRS took longer, resulted in smaller node yields, but had less blood loss, shorter hospital stays, and fewer intraoperative complications. However, the increased conversion rates to OS from LSC are significantly higher as well as the postoperative complication rates compared to DRS.

The most recent and largest study to date was published by Paley et al. at the Swedish Medical Center [7]. They prospectively reviewed their first 1,000 DRS cases, 377 of which were undergoing DRS for uterine cancer staging. They compared these 377 cases to 131 HCs undergoing OS for uterine cancer staging. Different surgeons at the same institution were performing the surgeries, and patient demographics were comparable between the groups. Similar to prior studies, DRS versus OS resulted in shorter hospital stays and less blood loss. Also, in their analyses, they broke up operative times between three time periods (first year, second year, and third year of experience) and found that operative time significantly decreased across these time cohorts, even while average BMI increased. The most recent cohort has averaged 207 minutes for completion of staging procedures that included pelvic and para-aortic lymphadenectomy. Also, the total complication rates were lower among DRS versus OS (6.4% versus 20.6%) and they had a conversion rate to OS of 3.5%, due to adhesive disease (4), inability to deliver the uterus (4), and unanticipated extrauterine spread requiring laparotomy for debulking (3). 

#### 6.1.1. Recommendations: Level 2++ Grade C

These studies suggest that DRS is preferable over OS for uterine cancer staging because of the decrease in blood loss, hospital stay, recovery time, and complication rate. DRS may be preferable over LSC for uterine cancer staging among women with a high probability of needing to convert to OS, such as obese women, women with larger uteri, or women with prior abdominal surgeries. DRS also may be preferable over LSC for surgeons with less LSC experience. Despite the aforementioned studies that support DRS, until long-term outcomes are evaluated, such as tumor recurrence rates, we cannot assume equivalence of the surgical modalities in regards to their oncologic outcomes. Furthermore, in the US where healthcare expenditure is rising with limited resources, we must consider costs as well. Barnett et al. have ently reported that LSC is more cost effective from both a societal and hospital perspective [100]. While DRS may be preferred and even advantageous over LSC and OS, we must proceed with caution before adopting DRS as common practice.

### 6.2. Cervical Cancer

Another common application of DRS in gynecologic oncology since its introduction has been in the surgical management of early stage cervical cancer. Worldwide cervical cancer is the second most common cancer among women. Fortunately in the US the rates of cervical cancer have declined significantly over the last 50 years due to the advent and adoption of routine pap smears. However, many women are still affected by cervical cancer in the US with approximately 493,000 new cases and 274,000 deaths reported in 2002 [***]. For many decades, women with early cervical cancer have been managed with abdominal radical hysterectomies with excellent cure rates. However, with the introduction of laparoscopy, many gynecologic oncologists have begun utilizing LSC to perform radical hysterectomies. Several studies have shown decreased blood loss, hospital stay, recovery times, and increased cosmesis and quality of life by performing LSC radical hysterectomies (LRHs) compared to traditional abdominal radical hysterectomies (ARHs) [36, 101, 102].However, similar to LSC for uterine cancer staging, LSC for radical hysterectomies can be technically challenging for surgeons not experienced with LSC.Radical hysterectomies are technically challenging even in an open setting due to the extensive ureterolysis required and dissection around the bladder and rectum in order to resect the parametrium and uterosacral ligaments with the uterus and cervix. Hence, DRS offers better control and visualization over LSC during this complex procedure. The first reported case of robotic radical hysterectomies (RRH) was by March et al. who reported 30 gynecologic cases using DRS, 7 of which were type II radical hysterectomies for stage I cervical cancer [50]. Subsequently Sert and Albert published a case report of a type III radical hysterectomy [103] followed by a comparative case series of their first 7 RRHs compared to 8 LRHs. They reported no difference in operative times or node yield and size of parametrial tissue excised, but bleeding and hospital stays were shorter for RRH [104]. These studies were followed by several additional case series [105–108]. Here we will focus on comparative studies meeting the aforementioned inclusion and exclusion criteria. For a more complete and comprehensive review of the literature, see the recent report of “Role of Robot-Assisted Surgery in Cervical Cancer,” by Yim et al. [109]. 

Soon after Sert and Albert published their comparative data on RRH [104], Magrina et al. at the Mayo Clinic in Arizona published their experience with RRH compared to ARH and LRH [92]. The 27 RRH cases included 18 done for cervical cancer and 9 done for endometrial cancer. They matched these cases to ARH and LRH cases with similar age, BMI, malignancy, stage, and type of radical hysterectomy. They found longer operative times for LRH compared to RRH, but RRH and ARH were equivalent. Blood loss was less for RRH compared to ARH, but RRH and LRH were not significantly different. Also, complication rates and node yield were equivalent across all modalities, and there were no conversions to OS. Furthermore, with an average followup of 31 months, none of the 18 patients with cervical cancer had recurred. Thus, they concluded that robotic and laparoscopic procedures were preferable over open surgery for radical hysterectomies [92]. 

Boggess et al. then published their outcomes of their first 51 consecutive cases of type III RRH compared to 49 cases of type III ARH for early cervical cancer done prior to initiation of the robotics program [85]. These patients were matched based on cancer type and stage. The groups were comparable in BMI and uterine size, but the RRH group was significantly younger and more likely to have had prior abdominal surgery (51% versus 18%). Still, the RRH group had significantly shorter operative times, less blood loss, shorter hospital stays, and higher node yields than the ARH group. Lastly, complication rates were equivalent. Again, this study suggests the superiority of RRH over ARH. 

The next two comparative studies included 12 and 16 patients in the RRH groups and thus were excluded from this review, but nonetheless concluded that RRH was likely advantageous over ARH from their case-control outcome data [110, 111]. Geisler et al. then published their experience with their first 30 cases of type III RRH compared to 30 previous cases of type III ARH [93]. Patients were comparable in age and BMI. They found decreased blood loss and length of hospital stay among the RRH group, but increased cases of urinary retention among RRH compared to ARH. However, operative time, node yield, and size of parametrial tissue were equivalent. They also found higher rates of urinary retention up to 1 month after surgery in the RRH group, but by 90 days there was no difference. 

Estape et al. at South Miami Hospital published their experience with 32 RRH cases compared to 17 and 14 stage and tumor-type matched cases of LRH and ARH [94]. The same surgeon performed all the LRH and ARH cases, and two surgeons performed all the RRH cases. Patients were comparable, except that patients in the RRH were significantly older than ARH patients. Still, RRH was faster and had improved blood loss, duration of hospital stays, time to return to work, with higher node yields compared to ARH. Also, RRH was equivalent to ARH in regards to surgical margin status and complication rates, but there was a trend towards decreased complications among RRH compared to ARH (19% versus 29%). RRH also appeared equivalent to LRH in regards to operative times, blood loss, and hospital stay, but resulted in higher node yields. And again, there was no significant difference in complication rates, but a trend towards decreased complications with RRH compared to LRH (19% versus 24%). They also looked at survival status among the three groups, and there were no differences, but due to drastic differences in follow-up times, it is difficult to make any conclusions from their survival rates. They concluded that RRH was superior to both ARH and LRH [94]. 

Whereas the aforementioned studies all suggest a benefit of RRH over ARH, LRH appears equivalent or inferior to RRH in regards to operative times, node yield, or complication rates. However, Nezhat et al. published a comparative case series of type III RRH to LRH and found equivalent outcomes in regards to operative time, blood loss, hospital stay, complication rates, and node yield between the two modalities [112]. Experienced laparoscopic surgeons performed all of the procedures, and the groups were comparable in regards to demographics and tumor characteristics. They also reported no recurrences in a mean follow-up time of 12 months and 29 months in the RRH and LRH groups. However, due to their small sample size (*n* = 17 in the RRH group) there is a high probability of a type II error, that is, LRH is indeed inferior to RRH, but such a difference was not detectable. Due to the small sample size this study was excluded from the tabulated systematic review. Although their findings suggest an equivalence of robotic and laparoscopy, the authors refrain from making that conclusion and astutely note:

…our evidence, as well as the evidence of others, supports robotic surgery as a more attractive option, both for the surgeon and the patient. However, questions remain, including whether the robot provides any additional benefits to a surgeon who is an experienced laparoscopist and comfortable performing the most advanced gynecologic procedures using traditional laparoscopy, whether there is an advantage for an inexperienced laparoscopic surgeon to use robotic technology compared with traditional laparoscopic instrumentation, and what the learning curve is with either approach [112].

The only other study that exclusively compared RRH to LRH has been recently published by Tinelli et al. [98] They compared 23 RRH to 76 LRH done at their center in Italy or Mount Sinai in New York for early stage cervical cancer. They excluded women with a BMI >35. Again, the groups were comparable. The only significant difference was a decreased operative time for RRH compared to LRH, otherwise all the outcomes were similar; and disease-free survival was 96% and 94% for the RRH and LRH patients respectively, a nonsignificant difference. Again, RRH appears equivalent to LRH in experienced hands but may still have advantages in decreasing operative times and complications.

Another study by Lambaudie et al. evaluated the use of OS, LSC, and DRS in managing locally advanced cervical cancer after chemoradiation [113]. Although the types of procedures done in each group are not clearly defined (lymph-node dissections versus simple hysterectomies versus radical hysterectomies), they found that DRS reduced hospital stays and had a lower rate of serious complications as compared to OS. However, like Nezhat et al., they found no difference in outcomes between DRS and LSC [112]. They thus concluded that, “despite the multiple advantages of robotic assistance published in the literature, robot-assisted procedures have no advantages compared to traditional laparoscopic radical hysterectomy when performed by an experience laparoscopic surgeon” [113].

The only other study published to date that compares RRH to LRH is by Sert and Albert from Norway [97]. However, this comparative case series includes only 7 cases of LRH. Unlike Nezhat et al. [112] and Tinelli et al. [98], they found that LRH had longer operative times, hospital stays, and higher blood loss compared to RRH. Although the groups were comparable, due to their small sample size, it is hard to draw any conclusions.

Sert and Albert also compared RRH to ARH, again the groups were comparable, and like the previous studies RRH was advantageous due to shorter operative times, hospital stays and decreased blood loss. However, they found that RRH resulted in significantly fewer pelvic lymph-node yields compared to ARH. They did not comment on the difference in complication rates, but it appears that the RRH had significantly fewer postoperative complications (11% versus 46%). They did report long-term clinical outcomes such as cervical cancer recurrences, and despite balanced tumor characteristics between the groups and shorter mean followup times in the RRH group (36 months) compared to both the LRH (56 months) and the ARH (70 months) groups, all the recurrences (5) were in the RRH group and none in the LRH or ARH group. Although the size of the parametrial tissue and vaginal edge were equivalent between groups, the striking difference in recurrence rates questions the adequacy of RRH in resecting disease [97]. However, with small sample sizes, this difference in recurrence rates is unlikely to be significant. More studies need to be done to assess long-term oncologic outcomes among these patients managed with different surgical modalities.

The last three studies identified are all case-control comparative analyses of RRH compared to ARH; however, one of these studies had a sample size of 14 and thus was excluded from the systematic review [114]. This study from the Netherlands nonetheless had comparable groups and found significantly less blood loss and shorter hospital stays among the RRH group with no difference in node yields. It took 9 hours to complete their first RRH case, but after only 14 cases the operative time was reduced to just 4 hours. This was similar to the 3 hours and 45 minutes average among their open cases. Again, followup times were shorter for the RRH group compared to the historic control ARH group, yet there were 2 recurrences in the RRH group of 14 patients, and 1 in the ARH group of 13 patients. One of these recurrences included a pelvic-side wall recurrence concurrent with a port site metastasis that was managed with resection and pelvic radiation. Maggioni et al. then published their experience in Italy with RRH compared to ARH [95]. The groups were comparable across demographics, comorbidities, and stage; however, patients in the RRH group were on average significantly younger (44 versus 50 years old). Like most previous studies, they found longer operative times for RRH, but less blood loss and shorter hospital stays. However, they found significantly fewer node yields and no significant difference in postoperative complication rates. They also noted no difference in bladder dysfunction postoperatively. Their long-term followup, although shorter for the RRH group, revealed an equivalent number of recurrences in both groups (5) with equal numbers of local and distant recurrences. One distant recurrence in the RRH group involved a port-site. They concluded that RRH is feasible and safe, but hesitated to say it was superior without further cost-benefit analyses and assessment of long-term oncologic outcomes. 

Lastly, Nam et al. from Korea compared 32 cases of RRH to 32 historic matched cases of ARH [96]. Unlike previous studies were demographics were “comparable,” here the patient demographics and tumor characteristics were almost identical due to matching. Also, the RRH cases were collected after one year of initiating the robotics program, hence eliminated effects from the early parts of the learning curve. They found no difference in operative times or complications, but RRH had decreased blood loss and length of hospital stay. Their long-term followup, which was less in the RRH group, revealed two recurrences in the RRH group versus none in the ARH group. Therefore, they state that RRH seems to be preferable over ARH, but they encourage that more long-term oncologic outcome data is taken and prospective randomized trials are completed before robotic surgery becomes routine in managing patients with cervical cancer. A phase 3 protocol comparing LRH or RRH to ARH has been published and completion of this trial and their results are anxiously awaited [115].

In addition to radical hysterectomies, the DRS has been utilized in several other procedures when managing cervical cancer. Radical trachelectomies with lymphadenectomy are an option for women with early stage (IA-IB1) cervical cancer who desire fertility preservation. Although several case reports [108, 116–118] and two case series [119, 120] are encouraging in regards to the feasibility and safety of DRS in performing radical trachelectomies, the sample size is limited to a total of 14 cases between all the studies, and long-term clinical outcomes such as recurrence rates and fertility rates are lacking. In addition to trachelectomies, DRS has also been described in performing radical parametrectomies in 5 patients [121] and a description of the surgical technique of DRS in uterine artery sparing radical trachelectomies [122], lymph-node dissections [123, 124], nerve-sparing radical hysterectomies [99], and even pelvic exenterations [125–127]. However, once again, these studies are either descriptions of techniques, case reports, or small case series with no comparative data. These studies suggest that DRS may be feasible in performing the aforementioned procedures, but further studies are needed before such surgical management approaches can be considered acceptable.

#### 6.2.1. Recommendations: Level 2++ Grade C

These studies, like those in uterine cancer staging, suggests that RRH is preferable over ARH due to its decrease in blood loss, hospital stay, recovery time, and complications. However, RRH appears to be equivalent to LRH in the hands of experienced surgeons. Again, if a patient is predicted to have a high chance for conversion to OS from LSC, even an experienced LSC surgeon may prefer DRS to prevent conversion and decrease complications. In addition, a less experienced LSC may prefer RRH to LRH. Once again, before the adoption of RRH into common practice for the management of early cervical cancer, further cost-effective analyses, evaluation of long-term oncologic outcomes, and the anticipated results from the phase III randomized control trial need to be critically assessed.

### 6.3. Ovarian Cancer

One in 72 women will develop epithelial ovarian cancer (EOC) over the course of their lifetime, and unfortunately, there is a dismal 5-year survival rate of only 46% [128]. EOC is staged surgically which for almost a century has involved an exploratory laparotomy with hysterectomy, adnexectomy, abdominal-pelvic washings, peritoneal biopsies, omentectomy, pelvic and para-aortic lymphadenectomy, and debulking of metastatic tumor when feasible with a goal of leaving no residual disease. For 85% of ovarian cancer patients with advanced stage disease these surgeries often require extensive dissections within the pelvis and upper abdomen and can require bowel surgery or at minimum mobilization of the bowel to adequately explore the pelvis and abdomen. Minimally invasive surgery can be very challenging in many of these cases. However, among the 15% of ovarian cancer patients with early stage disease confined to one or both ovaries or with extension to pelvic structures only, the utilization of LSC and DRS is now being considered. Unfortunately, this body of literature is still in its infancy including a couple case reports and case series [49, 111, 129] and one comparative case-control study [130]. A recent review article has reviewed these case reports and case series in addition to all the literature evaluating the use of MIS in staging EOC and borderline ovarian tumors [131]. They concluded:

There still are only limited data with regard to minimally invasive surgery for ovarian cancer; however, in the setting of [borderline ovarian tumors] BOTs and presumed early-stage EOC, minimally invasive surgical staging performed by a trained gynecologic oncologist appears to be safe and effective when compared to laparotomy. The minimally invasive approach allows for equivalent staging adequacy with fewer intraoperative complications and a shorter postoperative recovery [131].

Subsequent to this review, Magrina et al. compared 25 cases of DRS utilized for staging of EOC compared to 27 LSC cases and 119 OS cases done during the same period or in the 3 years prior matched by age, BMI, type and number of procedures done [130]. They also broke each cohort into subset by type of debulking. Type I included primary tumor excision including hysterectomy, adnexectomy, omentectomy, pelvic and para-aortic lymphadenectomy, appendectomy and the removal of peritoneal disease if present. Type II included the aforementioned procedures and one additional major procedure, while type III had an additional 2 or more major procedures. The majority of patients studied had advanced (stage III-IV) EOC: 60%, 75%, and 87% in the DRS, LSC, and OS groups, respectively. Although they do not mention if these differences in proportion are significantly different, they could bias the results of the study in favor or DRS and even LSC. This difference in stage may also affect the optimal (no visible disease) debulking rates reported between the cohorts: 84%, 93%, and 56% in the DRS, LSC, and OS groups, respectively. Also, approximately 25% of the patients in all the groups received neoadjuvant chemotherapy, making determination of surgical stage impossible among this subset of patients. Hence, there is potential for several biases due to the heterogeneity of patients within and between cohorts. Nonetheless, they did find decreased blood loss and hospital stay among DRS and LSC patients compared to OS patients, but there was no difference between DRS and LSC. Also, operative times were longer for DRS, but LSC and OS were similar. The only difference in perioperative outcomes was a significantly decreased post-operative complication rate among DRS and LSC patients that had type II debulkings. There was no significant difference among those having type I debulking surgeries, and among those getting type III, only 2 patients had DRS, and the remaining 32 had OS. Therefore, the numbers are too small for comparison. They did report long-term outcome data, both 3-year overall survival (OS) and 3-year progression-free survival (PFS) between the three surgical modalities and within subsets based on early and late stage disease. There was no difference in OS in early and late stage EOC and no difference in PFS in early stage EOC between all three surgical modalities. However, there was a significantly different 3-year PFS among DRS, LSC, and OS at 56%, 49%, and 30%, respectively (*P* = 0.03). Although *P* < 0.05, due to the multiple log-rank tests done with no adjustment for multiplicity, this is likely a type I error. Based on their findings they conclude that DRS and LSC are preferable over OS for patients requiring a type II ovarian debulking procedure and that survival was unaffected by surgical approach (see Table 3). 

#### 6.3.1. Recommendations: Level 2− Grade D

The prior study suggests at least equivalence of DRS and LSC compared to OS for EOC debulking with one or no additional major procedures, but due to the many potential confounding variables, no conclusions or final recommendations can be given from this single study. Also, there is not enough data to support the feasibility and safety of DRS in debulking EOC in cases that require at least 2 additional major procedures. Furthermore, there is not enough data to support the equivalency in terms of progression free survival or overall survival and it is doubtful that a minimally invasive approach would offer an equal opportunity for a surgeon to perform an optimal cytoreduction when compared to an open approach. Therefore it is not advised to perform robotic-assisted surgery for the diagnosis and management of advanced (stage III-IV) ovarian cancer outside of study protocol.

## 7. Conclusions

Robotic surgery offers many new advantages in the field of gynecology. Among general and reproductive gynecology as well as urogynecology, surgeries that would have traditionally been done as an exploratory laparotomy may now be done robotically, thus, avoiding the morbidity associated with an open surgery. In the field of gynecologic oncology, surgeries that have traditionally been done with exploratory laparotomy, are now being done with the DRS. Nonetheless, many of these procedures in benign and oncologic gynecology, may be completed with conventional laparoscopy as well in the hands of an experienced laparoscopic surgeon. However, even with an experienced and skilled surgeon, obese patients, patients with prior abdominal surgeries, or patients requiring lengthy and high-risk surgeries likely benefit from DRS over LSC. Thus, each case must be considered individually with careful attention to the disease, the patient and their comorbidities and goals, the skill and confidence of the surgeon, and the practice setting. For example, DRS may be more feasible and cost effective in a hospital equipped with a routinely used DRS that also has well-trained staff that can assist and troubleshoot the device as needed. 

However, as mentioned previously, many of the disease states for which DRS is being used have limited long-term outcome data, which is very important in regards to oncologic outcomes and urogynecologic outcomes. Also, all the outcomes discussed are from comparative observational studies, with a variety of methodologies and patient characteristics. Thus, the results cannot be generalized to all patients, but rather must be considered in the context in which the studies were done and applied to individual scenarios only when appropriate. Until larger, well-designed observational studies or randomized control trials are completed which also report long-term outcomes, we cannot definitively state the superiority of DRS over other surgical methods; although, the majority studies do suggest DRS is preferred over OS and possibly LSC. 

With any new innovation comes great potential for progress, but also harm. This notion applies to DRS. With the right amount of training and skill, along with appropriate patient selection, DRS can be highly advantageous. Patients will likely have less blood loss, less post-operative pain, faster recoveries, and fewer complications compared to OS and potentially even LSC. Yet, in agreement with the ACOG Technology Assessment of “Robot-Assisted Surgery” in 2009 [132], further studies as well as additional cost-effective analyses need to be done to critically evaluate the role of robotic surgery in gynecology before it is adopted as common practice in managing gynecologic diseases. 

## Figures and Tables

**Table 1:** Comparative observational studies evaluating the da vinci robotic system (DRS) versus open surgery (OS) or laparoscopy (LSC) to perform general gynecologic as well as reproductive and urogynecologic procedures.

Surgery	N	DRS versus OS	DRS versus LSC	Control	Surg-eon	OR time (min.)	EBL (mL)	Hospital stay (days)	Conversions to OS	Bld Tx	Intraoperative complications	Postoperative complications	Clinical outcomes
*Adnexectomy*													
Margina et al. [24] 2008	85 versus 91		*✓*	CC	SS	**77 versus 62** ** (median)**	25 versus 50 (median)	0 versus 0 (median)	0	0	1/85 versus 2/90 uretral injury (1) versus uretral injury (1) and rectotomy (1)	Major: none Minor:10/81 versus 10/91	—

*Myomectomy*													
Advincula et al. [25] 2000–2004	29 versus 29	*✓*		CC matched	DS	**231 versus 154**	**196 versus 365**	**1.5 versus 3.6**	0	0 versus 2	1/29 versus 0/29cardiogenic shot from vasopressin (1)	Major: 0 versus 3arrest, DVT, ARF in same pt. (1), bld tx (2) Minor: 3/29 versus 11/29	—

Nezhat et al. [18] 2006-2007	15 versus 35		*✓*	CC matched	SS	**234 versus 203**	370 versus 420	1 versus 1.1	0	0	none	Major: none Minor: NR	6.7% versus 7.5% pregnancy rate

Bedient et al. [26] 2000–2008	40 versus 41		*✓*	CC adjusted	—	144 versus 161	100 versus 250	>2 days:12% versus 23%	0 versus 5%	2 versus 2	1/41 versus 8/40bleeding (1) versus bleeding (6), conversion (2)	Major: 4 versus 2Bld tx (2), bowel injury (1), pelvic abscess (1) versus bld tx (2) Minor: 3 versus 4	85% versus 83% symptom resolution

Ascher-Walsh and Capes [27] 2005–2008	75 versus 50	*✓*		HC(≤3 myomas)	—	**192 versus 138**	**226 versus 459**	**0.5 versus 3.3**	0	0	none	Major: none Minor: 2/75 versus 19/50febrile morbidity 1 versus 19	—

Barakat [28] 2008-2009	89 versus 393 89 versus 93	*✓*	*✓*	HC HC	__ __	**181 versus 126**	181 versus 155	**1 versus 3** 1 versus 1 (median)	0 0 versus 1	**DRS: 0** **LSC: 2** **OS: 25**	DRS (0), LSC (1) (bowel injury), OS (0) Intraoperative hemorrhage: NR	Major: DRS (0), LSC (0), OS (1) (wound separation) Minor: DRS (0), LSC (1), OS (0)	—

*Hysterectomy*													
Payne and Dauterive [29] 2006-2007	100 versus 100		*✓* LSC = Type IVE	HC	SS	**119 versus 92**	**61 versus 113**	**1 versus 1.6**	**4% versus 9%**	0	1/100 versus 2/100 cystotomy (1) versus enterotomy (1), vaginal tear (1)	Major: none Minor:1/100 versus 0	—

Shashoua et al. [30] (2005–2007)	24 versus 44		*✓* LSC = Type IVE	HC	SS	**142 versus 122**	1.9 versus 1.8 (hgb drop)	**1.0 versus 1.4**	0	0	none	Major: 0 versus 1, cuff dehiscence Minor: 1/24 versus 0	—

Sarlos et al. [31] 2007–2009	40 versus 150		*✓* LSC = TLH	HC matched	SS	**109 versus 83**	<50 versus 81	3.3 versus 3.9	0	0	none	Major: none Minor: 5/40 versus 0	—

Giep et al. [32] 2007–2009	237 versus 265		*✓* LSC = LAVH	CC & HC	DS	**90 versus 125**	59 versus 168	**1.0 versus 1.2**	1.7% versus 0.4%	0	1/237 versus 1/265cystotomies	Major: 2 versus 1 PE (1), abscess (1) versus abscess (1) Minor: 6/237 versus 3/265	—

*Sacrocolpopexy*													
Geller et al. [33] 2004–2008	73 versus 105	*✓*		CC	DS	**328 versus 225**	103 versus 255	**1.3 versus 2.7**	1.4% versus 0	1 versus 4	1/73 versus 1/105 cystotomies	Major: 1 versus 0 PE Minor: 11/73 versus 11/105	Improvement in POP-Q C point at 6 m f/u

Historic controls (HCs), Concurrent controls (CCs), same surgeon(s) (SS), different surgeon(s) (DS), estimated blood loss (EBL), not reported (NR). Sample size (N). When not specified, DRS outcomes are reported first. Items in **bold** are **significantly different** determined by a two-sided alpha <0.05. Outcomes are reported as means unless otherwise noted.

**Table 2:** Comparative observational studies evaluating the da vinci robotic system (DRS) versus open surgery (OS) or laparoscopy (LSC) to perform uterine cancer staging procedures.

Surgery	N	DRS versus OS	DRS versus LSC	Control	Surgeon	OR time (min.)	EBL (mL)	Hospital stay (days)	Lymph node count	Conversions	Bld Tx	Intraoperative complications	Postoperative complications
*Uterine Cancer Staging*													
Veljovich et al. [83] 2006-2007 Swedish Medical CenterSeattle, WA	25 versus 131 25 versus 4	*✓*	*✓*	HC + CC Pro-spective HC + CC	DS	**283 versus 139** 283 versus 255	**67 versus 198** 67 versus 75	**1.7 versus 5.3** 1.7 versus 1.2	18 versus 13 18 versus 20	DRS: 1	NR	NR	DRS: Major: 8% Infection (1), cuff dehiscence (1) Minor: 12% OS: Major 21% Cardiac (5), pulmonary (5), renal (4), CVA (1), infection (3), wound dehiscence (9) Minor: 8% LSC: NR

Gehrig et al. [84] 2005–2007 BMI ≥ 30 UNC	49 versus 32		*✓*	HC	NR	**189 versus 215**	**50 versus 150**	**1.0 versus 1.3**	**31 versus 24**	DRS: 0 LSC: 3	NR	DRS: Enterotomy (1) LSC: none	DRS: Major: 8%port-site hernia (3), cuff complication (1) Minor 2% LSC: Major 6% port-site hernia (1), cuff complication (1) Minor 3%

Boggess et al. [85] 2005–2007 UNC	103 versus 138 103 versus 81	*✓*	*✓*	HC HC	DS SS	**191 versus 146** **191 versus 213**	**75 versus 266** **75 versus 146**	**1 versus 4.4** **1 versus 1.2**	**33 versus 15** **33 versus 23**	DRS: 2.8% LSC: 3.7%	DRS: 1% OS: 1.4% LSC: 2.5%	DRS: bowel injury (1) OS: enterotomy (1) LSC:caval injury (1), bowel injury (1)	**DRS: Major: 2% ** Port-site hernia (1), PE (1) Minor: 3% **OS: Major: 8%** CVA (1), ileus + readmission (7), DVT (2), PE (1), respiratory failure (1) Minor: 21% LSC: Major: 5% port-site hernia (1), ileus readmission (1), vaginal dehiscence (1), abscess (1) Minor: 5%

Bell et al. [86] 2005–2007 Sanford Clinic, Sioux Falls, SD	40 versus 40 40 versus 30	*✓*	*✓*	HC + CC Retro-spective HC + CC	SS SS	**184 versus 209** 184 versus 171	**166 versus 316** 166 versus 253	**2.3 versus 4.0** 2.3 versus 4.0	17 versus 14 17 versus 17	NR	DRS: 5% OS: 15% LSC: 10%	DRS: None OS: genital femoral n. damage (1) LSC: caval injury (1)	**DRS: 8%** Port-site hernia (1), re-operation for bleeding (1), delayed void (1) **OS: 25%** Ileus (5), wound infection (2), lymphedema (1), cuff hematoma (1), incisional hernia (1) **LSC: 23%** Wound infection (3), DVT (1), cuff dehiscence (1), superficial phlebitis (1), A-fib (1)

DeNardis et al. [87] 2006-2007 Florida Hospital Cancer Institute	56 versus 106	*✓*		HC Retro-spective	DS	**177 versus 79**	**105 versus 241**	**1.0 versus 3.2**	19 versus 18	DRS: 5.4%	**0% versus 9%**	None	**DRS: 20%** respiratory failure (1), ileus (1), fever (2), atelectasis (1), wound complication (1), cuff hematoma/separation (4), UTI (1) **OS: 61%** fever (17), anemia (13), Ileus (4), ARF (2), PE (1), colitis (1), urinary retention (2), thrush (1), UTI (4), atelectasis (4), wound complication (13), lymphocele (1), cuff hematoma/separation (2)

Seamon et al. [88] 2006–2008 OU	105 versus 76		*✓*	HC Pro-spective	SS	**242 versus 287**	**100 versus 250**	**1 versus 2 (med.)**	31 versus 33	**DRS: 12% ** **LSC: 26%**	**3% versus 13%**	3 versus 2 DRS: vessel injury (1), GI injury (2) versus OS: nerve injury (1),urinary tract injury (1)	DRS: 9% port-site herniation (1), cardiac (1), pulmonary (1), other (5) LSC: 10% DVT (1), cardiac (1), neurologic (1), other (3)

Seamon et al. [89] 2006 –2008 BMI ≥ 30 OU + UAB Matched: BMI + surgeon 1 : 2	109 versus 191	*✓*		HC Retro- spective	SS	**228 versus 143**	**109 versus 394**	**1 versus 3**	25 versus 24	DRS: 15.6%	**2% versus 9%**	1 versus 2 DRS: GI injury (1) versus OS: vessel injury (1), GI injury (1)	**DRS: 10% ** cardiac (1), pulmonary (2), GI (2), urologic (1), fever (1), fistula (1), bleeding (1), other (3) **OS: 26%** DVT (1), cardiac (2), pulmonary (2), GI (19), urologic (2), (neurologic (1), fever (4), ARF (3), opiated OD (1), parasthesia (2), arrest (1), death (1)Wound complications: **11% versus 27%**

Jung et al. [80] 2006–2009 Korea	28 versus 56 28 versus 25	*✓*	*✓*	CC CC Pro-specitve	SS SS	193 versus 187 193 versus 165	NR	**7.9 versus 10.8** 7.9 versus 7.7	21 versus 24 **21 versus 18**	none	DRS: 14% **OS: 43%** LSC: 16%	DRS: none OS: 1 vessel injury LSC: none	**DRS: 7%** pelvic infection (2) **OS: 23% ** uretral stricture (1), pelvic infection (2), ileus (1), incisional hernia (1), wound dehiscence (6), lymphocele (1), lymphedema (1) **LSC: 8% ** Pelvic infection (2)

Cardenas-Goicoechea et al. [90] 2007–2010 U Penn	102 versus 173		*✓*	HC Retro-spective	SS	**237 versus 178**	**109 versus 187**	1.9 versus 2.3	22 versus 23	DRS: 1% LSC: 5.2%	2.9% versus 1.7%	DRS: 2GI injury (2) LSC: 6 Urinary tract injury (6)Higher rates of uretral injury in LSC	DRS: Major (2%) PE (1), enterocutaneous fistula (1) DRS: Minor 8%: lymphocele (1), UTI (2), cuff dehiscence (1), abscess (2), incsional hernia (1), ileus (1) LSC: Major (0%) Minor 8%: lymphocele (3), UTI (1), Pna (2), wound seroma (2), cuff cellulitis (2), SBO (1), hematoma (1), port-site abscess (1)

Lim et al. [91] 2008-2009 Center of HopeReno, Nevada	56 versus 36 56 versus 56	*✓*	*✓*	HC HC Pro-spective	SS Initial exp SSInitial exp	**163 versus 137** **163 versus 192**	**89 versus 266** **89 versus 209**	**1.6 versus 4.9** **1.6 versus 2.6**	**27 versus 56** **27 versus 45**	DRS: 1.8% LSC:7.1%	DRS: 0% OS: 3% LSC: 0%	DRS: 0OS: 0 **LSC: 13% (7)** obturator n. injury (3), cystotomy (2), enterotomy (1), vessel injury (1)	DRS: Major: 5% PE (1), abscess (1), perforated gastric ulcer (1) Minor: 9% OS: Major: 14% Abscess/sepsis (1), fistula (1), pna (1), PE (1), hypertensive crisis (1) Minor: 3% LSC: Major: 11% Fistula (1), obturator palsy (3), bacteremia (1), DVT (1) Minor: 4%

Lim et al. [82] 2008–2010 Center of Hope Reno, Nevada	122 versus 122		*✓*	HC Pro-spective	SSInitial ex	**147 versus 187**	**81 versus 207**	**1.5 versus 3.2**	**25 versus 43**	**DRS: 0.8%** **LSC: 6.6%**	DRS: 0% LSC: 2.5%	**DRS: 0.8%** Enterotomy (1) **LSC: 6%** Obturator n. injury (3), cystotomy (2), enterotomy (1), vessel injury (1)	**DRS: Major: 4%** PE (1), abscess (1), perforated gastric ulcer (1), bowel perforation (1) Minor: 6% **LSC: Major: 10%** Fistula (1), obturator palsy (3), bacteremia (1), DVT (1), richter hernia (1), CVA (1) Minor: 2%

Paley et al. [7] 2006–2009 Swedish Medical CenterSeattle, WA	377 versus 131	*✓*		HC Pro-spective	DS	**184 versus 139**	**47 versus 198**	**1.4 versus 5.3**	**16 versus 13**	DRS: 3.5%	DRS: 0.5% OS: 0.8%	**DRS: 0.5%** Vessel injury (1), cystotomy (1) **OS: 3%** Uretral injury/ARF	**DRS: 5%** cardiac (1), pulmonary (3), DVT/PE (3), infection (4), labile bld sugar (2), ileus/SBO (2), chylous ascites (1), cuff dehiscence (4) **OS: 17%** Cardiac (5), pulmonary (1), DVT/PE (1), infection (6), wound dehiscence (9)

Hisoric conrols (HC), Concurrent controls (CC), same surgeon(s) (SS), different surgeon(s) (DS), estimated blood loss (EBL), not reported (NR). Sample size (N).

When not specified, DRS outcomes are reported first. Items in **bold** are **significantly different** determined by a two-sided alpha <0.05. Outcomes are reported as means unless otherwise noted.

**Table 3:** Comparative observational studies evaluating the da vinci robotic system (DRS) versus open surgery (OS) or Laparoscopy (LSC) to perform robotic radical hysterectomies (RRH), abdominal radical hysterectomies (ARH), or Laparoscopic radical hysterectomies (LRH) for cervical cancer and a comparative observational study of DRS versus OS and LSC to perform ovarian cancer staging and debulking surgeries.

Surgery	N	RRH versus ARH	RRH versus LRH	Control	Surg-eon	OR time (min.)	EBL (mL)	Hospital stay (days)	Lymph node count	Conversions	Bld Tx	Intraoperative complications	Postoperative complications
*Radical hysterectomy*													
Magrina [92] 2003–2006 Mayo ClinicPhoenix, AZ Matched: age, BMI, malignancy, stage, type of radical	27 versus 35 27 versus 31	*✓*	*✓*	HC + CC HC + CC Pro-Spective	NR	190 versus 167 **190 versus 220**	**133 versus 444** 133 versus 208	**1.7 versus 3.6** 1.7 versus 2.4	26 versus 28 26 versus 26	None	RRH: 4% ARH:9% LRH: 0%	RRH: 0 ARH: 6% Bleeding (2) LRH: 3% Rectotomy (1)	RRH: Major 7% Pleural effusions (1), pneumothorax (1) Minor: 15% urinary retention (1), UTI (2), bleeding (1) ARH: Major 9% Pna (1), wound seroma (1), ileus (1)Minor: 9% urinary retention (1), emesis (2) LRH: Major 6% Fever (1), port-site infection (1) Minor: 10% corneal abrasion (1), urinary retention (1), lymphatic drainage at cuff (1)

Boggess et al. [81] 2005–2007 UNC Matched: stage	51 versus 49	*✓*		HC Pro-Spective	NR	**211 versus 248**	**97 versus 417**	**1 versus 3.2**	**34 versus 23**	None	RRH: 0% ARH:8%	NR	RRH: 8% Cuff abscess (1), lymphedema (1), readmission (1), cuff dehiscence (1) ARH: 16% Femoral n. injury (2), ileus (1), lymphocyst (1), bleeding (1), cuff dehiscence (1), wound infection (2)

Geisler [93] 2007-2008 Toledo, OH	30 versus 30	*✓*		HC Pro-spective	NR	154 versus 166	**165 versus 323**	**1.4 versus 2.8**	25 versus 26	none	NR	NR	Urinary retention POD no. 8 RRH: 27% ARH: 3% 90 days post-op: ND

Estape [94] 2006–2008 South Miami Hospital Matched: stage and histology	32 versus 14 32 versus 17	*✓*	*✓*	HC HC Pro-spective	DS	**144 versus 114** 144 versus 132	**130 versus 621** 130 versus 210	**2.6 versus 4.0** 2.6 versus 2.3	**32 versus 26** **32 versus 19**	NR	RRH: 3% ARH: 36% LRH: 0%	RRH: 3% Cystotomy (1) ARH: 0% LRH: 12% Cystotomy (2)	RRH: 19% Atelectasis/COPD (1), fever (1), ileus (1), wound cellulitis (1), pelvic abscess (1), cuff dehiscence (1)ARH: 29% Pna (1), SVT (1), ureter dilation (1), urine retention (1) LRH: 24% Fever (1), hypo-K (1), ileus (1), fistula (1)

Maggioni et al. [95] 2007–2009 Milan, Italy	40 versus 40	*✓*		HC Pro-spective	DS	**272 versus 200**	**78 versus 221**	**3.7 versus 5.0**	**20 versus 26**	NR	RRH: 8% ARH: 23%	RRH: 5% Nerve injury (1), intestinal injury (1) ARH: 10% Cystotomy (1) ureteral injury (2), intestinal injury (2)	RRH: 30% Subcutaneous emphysema (4), fever (3), infection (1), nerve palsy (2), pleural effusion (1), reintervention (1) ARH: 25% Fever (12), infection (3), ileus (1), nerve palsy (3), pleural effusion (2), reintervention (1)

Nam et al. [96] 2006–2009 Seoul, Korea Matched: Age, BMI, stage, histology, prior surgeries, radical type	32 versus 32	*✓*		HC Pro-Spective	DS	218 versus 209	**220 versus 532**	**11.6 versus 16.9**	20 versus 24	NR	**RRH:** **3%** **ARH:** **64%**	RRH: 3% Rectotomy ARH: 0%	RRH: 84% Infection (2), fever (7), pleural effusion (2), bladder dysfunction (5), ileus (4), diarrhea (1), wound dehiscence (1), neuropathy (1), lymphedema (1), readmit (2), ureteral stricture (1)ARH: 59% Infection (1), fever (9), pleural effusion (1), bladder dysfunction (3), ileus (2), wound dehiscence (3)

Sert and Albert [97] 2005–2009 Norway	35 versus 26 35 versus 7	*✓*	*✓*	HC HC Pro-specitve	NR	**263 versus 163** **263 versus 364**	**82 versus 595** **82 versus 164**	**3.8 versus 9.2** **3.8 versus 8.4**	**20 versus 26** 20 versus 15	NR	NR	RRH: 9% Cystotomy (3)ARH: 0 LRH: 14% Cystotomy (1)	RRH: 11%Lymphocyst (2), lymphedema (1), DVT (1) ARH: 46%Lymphocyst (1), lymphedema (2), UTI (7), pna (2), LRH: 71%Lymphocyst (3), UTI (1), compartment syndrome (1)

Tinelli et al. [98] 2003–2010 Avellino, Italy & Mount Sinai, New York, NY	23 versus 76		*✓*	CC Pro-spective	DS	**255 versus 323**	95 versus 157	4 versus 3	Pelvic: 27 versus 25 Aortic:12 versus 10	none	0	RRH: 9% Cystotomy (2) LRH: 3% Cystotomy (2)	RRH: 9% Lymphorrhea (2), fever (2) LRH: 21% Fistula (1), fever (6), lymphorrhea (9)

*Ovarian cancer debulking*													
Magrina et al. [99] 2004–2008 Mayo Clinic Phoenix, AZ Matched: age, BMI, type debulking	25 versus 119 25 versus 27	*✓*	*✓*	HC + CC Pro-spective HC + CC	NR	**315 versus 261** **315 versus 254**	**164 versus 1307** 164 versus 267	**4.2 versus 9.4** 4.2 versus 3.2	25 versus 23 25 versus 21	NR	NR	DRS: 12% Cystotomy (2), vessel injury (1) OS: 13% Bleeding (6), GI injury (6), cystotomy (2), ureteral injury (2) LSC: 11% Enterotomy (1), bleeding (1), vessel injury (1)	DRS: 24% Cuf dehiscence (2), pleural effusion (1) bleeding (1), ileus (1), trocar-site infection (1), pulmonary edema (1) OS: 34% Wound complications (10), GI (11), CV (7), pulmonary (7), UTI(2), abscess (2), hematoma (1), anastamotic leak → death (1) LSC: 4% Pelvic abscess (1)

Hisoric conrols (HC), Concurrent controls (CC), same surgeon(s) (SS), different surgeon(s) (DS), estimated blood loss (EBL), not reported (NR). Sample size (N). When not specified, DRS outcomes are reported first. Items in **bold** are **significantly different** determined by a two-sided alpha <0.05. Outcomes are reported as means unless otherwise noted.
